# The clinical result of arthroscopic bone grafting and percutaneous K-wires fixation for management of scaphoid nonunions

**DOI:** 10.1097/MD.0000000000009987

**Published:** 2018-03-30

**Authors:** Young-Keun Lee, Kwang-Wook Choi, Sang-Hyun Woo, Pak Cheong Ho, Malrey Lee

**Affiliations:** a Department of Orthopedic Surgery, Research Institute of Clinical Medicine of Chonbuk National University – Biomedical Research Institute of Chonbuk National University Hospital, Jeonju, Chonbuk; b Department of Orthopedic Surgery, Research Institute of Clinical Medicine of Chonbuk National University—Biomedical Research Institute of Chonbuk National University Hospital, Jeonju; c W Institute for Hand and Reconstructive Microsurgery, W Hospital, Daegu, Republic of Korea; d Department of Orthopaedic and Traumatology, Prince of Wales Hospital, Shatin, N.T., Hong Kong; e The Research Center for Advanced Image and Information Technology, School of Electronics & Information Engineering, Chonbuk National University, JeonJu, Chonbuk, Republic of Korea.

**Keywords:** arthroscopy, bone graft, K-wire, scaphoid nonunion

## Abstract

The purpose of this study is to analyze the clinical results of patients with scaphoid nonunion treated with arthroscopic bone grafting and K (Kirschner)-wires fixation.

We retrospectively reviewed the records of 27 patients with scaphoid nonunion who had been treated with arthroscopic bone grafting and K-wires fixation method from November 2008 to February 2014. The average patient age was 35 years. The time from injury to treatment averaged 45 months. The average follow-up period was 18 months. Bone union was assessed using serial plain radiographs. The functional outcome was evaluated by comparing the modified Mayo wrist score with the visual analog scale (VAS) for pain, which were measured at the time of preoperation and at final follow-up.

Union was achieved in 26 of the 27 nonunions (96.29%). The average radiologic union time was 10 weeks. The average VAS score decreased from 6.38 (range, 3–10) preoperatively to 1.59 (range, 0–3) at the final follow-up. The average modified Mayo wrist score improved from 60.19 preoperatively to 83.46 at the final follow-up. According to this score, there were 12 excellent, 6 good, and 9 fair results at the final follow-up.

Arthroscopic bone grafting and percutaneous K-wires fixation is an effective treatment method for a scaphoid nonunion and has the advantages of allowing thorough assessment, enabling a comprehensive management approach for scaphoid nonunion in a minimally invasive manner, and this method can also be used for the scaphoid nonunion with SNAC stage I.

## 1 Introduction

Many surgical techniques have been developed to treat scaphoid nonunion such as corticocancellous or cancellous bone graft and various vascularized bone grafting techniques.^[1–3]^ Various studies have reported variable but somewhat successful results with the open grafting procedure with failure rates of 25% to 45%.^[4–6]^ For example, open grafting with dissection of wrist capsule and ligaments damages the joint and hence leads to increased stiffness of the wrist and hand. Additional surgical trauma may also jeopardize blood supply to the carpal bones.^[7,8]^ These features continuously challenge surgeons to devise new treatment procedures.

Slade et al^[9]^ first described the successful arthroscopy and percutaneous bone graft technique for scaphoid nonunion. Arthroscopic-assisted bone grafting and percutaneous fixation have advantages of minimal surgical trauma to the scaphoid blood supply and its ligament connection and provide a thorough wrist assessment, comprehensive approach for scaphoid nonunion and its sequelae in a minimally invasive manner.^[10–16]^ The purpose of this study is to analyze the clinical results of patients with scaphoid nonunion treated with arthroscopic bone grafting and K (Kirschner)-wires fixation.

## 2 Materials and methods

We retrospectively reviewed the records of 27 patients with scaphoid nonunion who had been treated with arthroscopic bone grafting and K-wires fixation method from November 2008 to February 2014. The patients included 26 males and 1 female with an average age of 35 years (range, 15–61 years). A total of 21 mid-third and 6 proximal third fractures were included. The time interval between injury and operation ranged from 3 months to 40 years (average 45 months). The most common causes of injuries were sports injuries in 12 patients, falls in 8 patients, traffic accidents in 2 patients, and unknown in 5 patients. The average follow-up period was 18 months (range, 12–49 months).

We classified scaphoid nonunion according to the Slade and Geissler's classification^[9]^ (Table 1), based on the radiologic and arthroscopic findings: 5 patients with grade 2, 9 patients with grade 3, 7 patients with grade 4, and 6 patients with grade 6. During arthroscopy, carpal ligament injuries were graded using the system described by Geissler et al^[17]^ and the lesions of the triangular fibrocartilage complex (TFCC) were classified according to the system described by Palmer.^[18]^

**Table 1:**
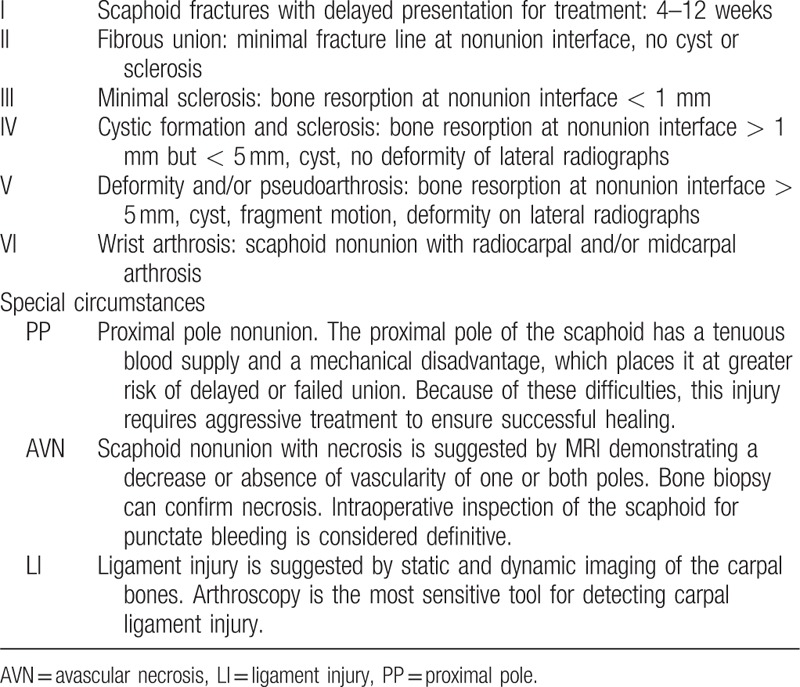
Treatment classification system for scaphoid nonunion^[9]^.

We identified scapholunate (SL) ligament injuries in 17 patients, of which 15 were classified as grade-II and 2 as grade-III. We identified grade II lunotriquetral (LT) ligament injuries in 3 patients and 3 were type I-A injuries of the TFCC. We identified scaphoid nonunion advanced collapse (SNAC) in 9 patients, all were in stage I. We identified punctate bleeding in both ends of the nonunion site during the arthroscopic debridement and we could not find punctate bleeding at the proximal fragment in 3 patients. We identified punctate bleeding in both ends of the nonunion in 24 wrists. In the remaining 3 wrists there was avascular necrosis (AVN) according to Green's prone viability test (Table 2).^[19]^

**Table 2:**
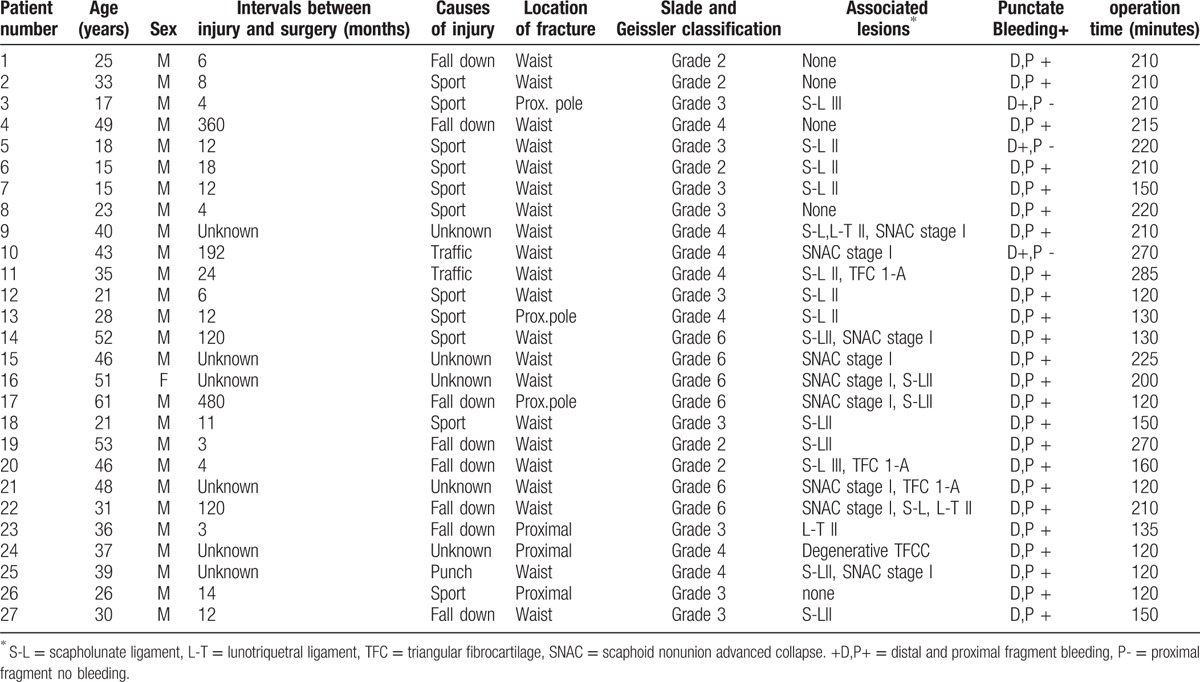
Demography of the patients.

Bone union was clinically assessed as the absence of tenderness at the anatomical snuffbox and radiologically with wrist posteroanterior (PA), lateral, semipronated oblique, and PA with ulnar deviation view assessed as the disappearance of the fracture line with bony trabecular across the original fracture. Patients were evaluated for range of motion (ROM), pinch strength, and grip strength measured on a dynamometer.^[20]^ Functional outcome was evaluated from a comparison between the modified Mayo wrist score and the visual analog scale (VAS) for pain (0 = no pain, 10 = worst pain), which were measured at the time of preoperation and final follow-up (Tables 2 and 3).^[21]^

**Table 3:**
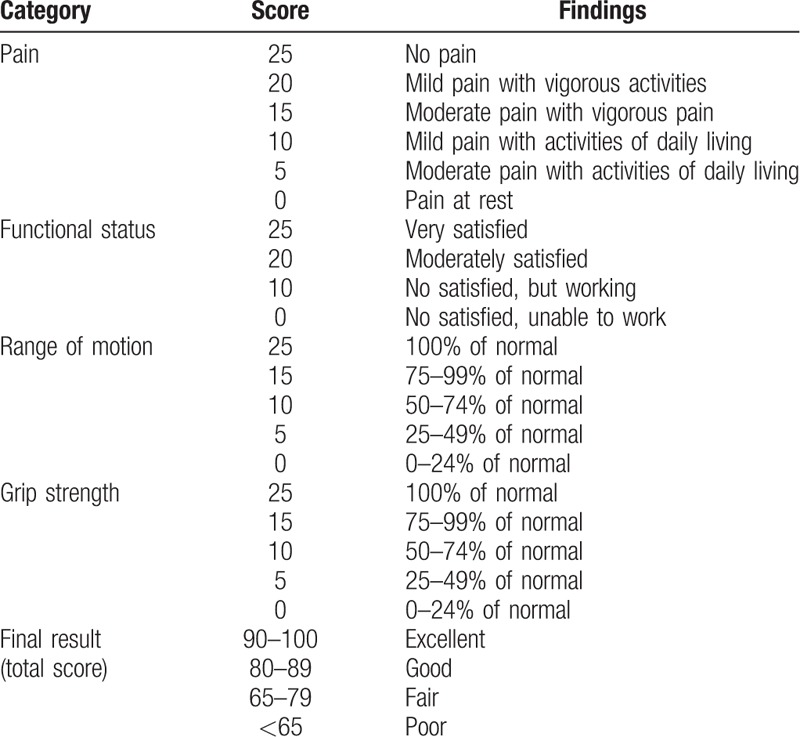
Modified Mayo wrist scoring system^[18]^.

### 2.1 Statistical analysis

IBM SPSS version 20.0 (IBM Corp., Armonk, NY) program was used for the statistical analysis of data. We used a Wilcoxon signed rank test to compare the preoperative and last follow-up ROM, grip and pinch strength, and modified Mayo wrist score changes. We used a Mann–Whitney *U*-test to compare the early and recent 10 cases’ operation times, respectively, and also to compare the results from whether SNAC exists or not. When the *P* value was < .05, it was considered statistically significant.

### 2.2 Surgical technique

The operation is performed under general anesthesia and the patient is positioned supine with the contralateral side of the iliac crest region draped for bone graft harvesting. The operated arm is placed in a wrist traction tower and a vertical traction of 4–6 kg force is applied through plastic finger trap devices to the middle 3 fingers for joint distraction on a hand table. An arm tourniquet is not applied and a C-arm image intensifier is prepared for the percutaneous scaphoid fracture reduction and K-wires fixation.

**Figure 1:**
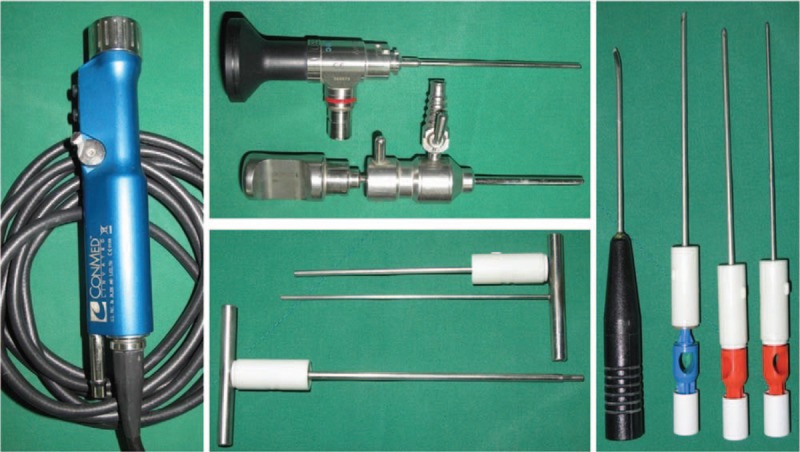
Basic instrumentation. 2.7 mm video arthroscope, 2.0 and 2.9 mm shavers, 3.0 mm burr and radiofrequency probe for surgical instrument. Two custom-made cannulas (3.8 and 3.0 mm) and 2 custom-made trocars (3.2 and 2.7 mm) for percutaneous bone grafting.

**Figure 2:**
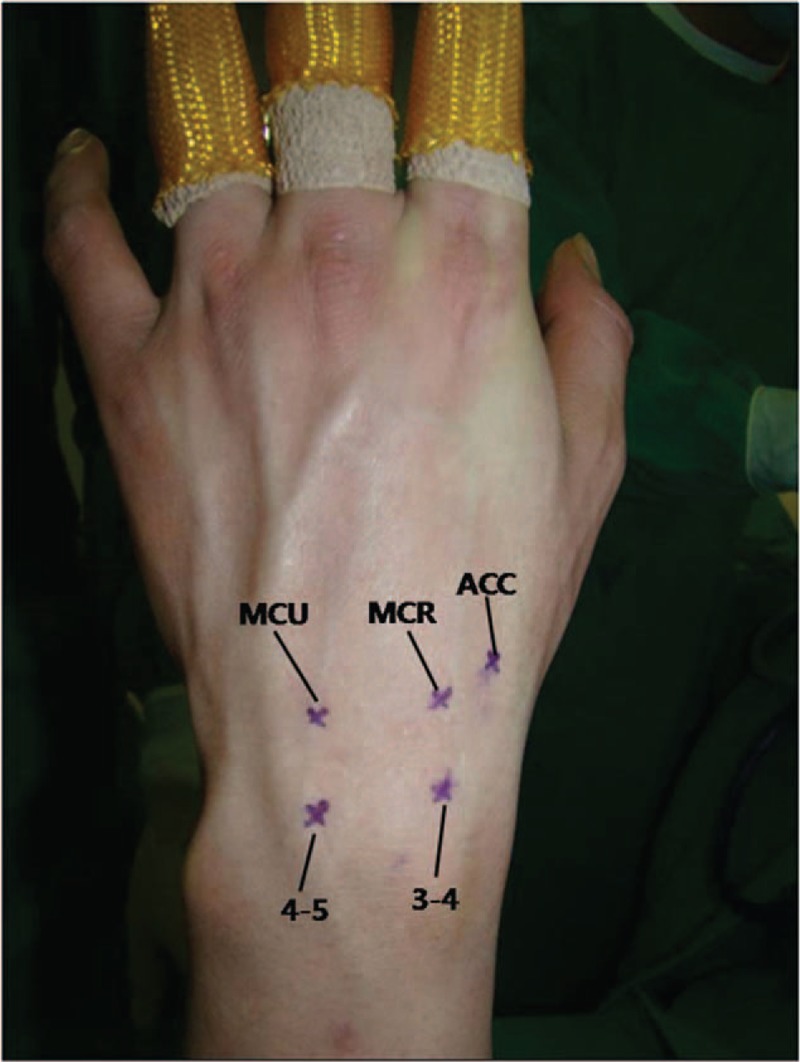
Arthroscopic portals marked in the radiocarpal and midcarpal joints. ACC = accessory portal, MCR =  midcarpal radial, MCU = midcarpal ulnar.

We use a 2.5 mm video arthroscope (Linvatec, CONMED Linvatec, Utica, NY), 2.0 and 2.9 mm shavers, a 3.0 mm burr, and a radiofrequency probe for surgical instruments. We also use 2 custom-made cannulas (3.8 and 3.0 mm) and 2 custom-made trocars (3.2 and 2.7 mm) for percutaneous bone grafting (Fig. 1). We employ continuous sending irrigation. We make 3/4 and 4/5 portals for the radio-carpal joint, and mid-carpal radial (MCR), mid-carpal ulna (MCU), and one accessory portal for the mid-carpal joint (Fig. 2).

**Figure 3:**
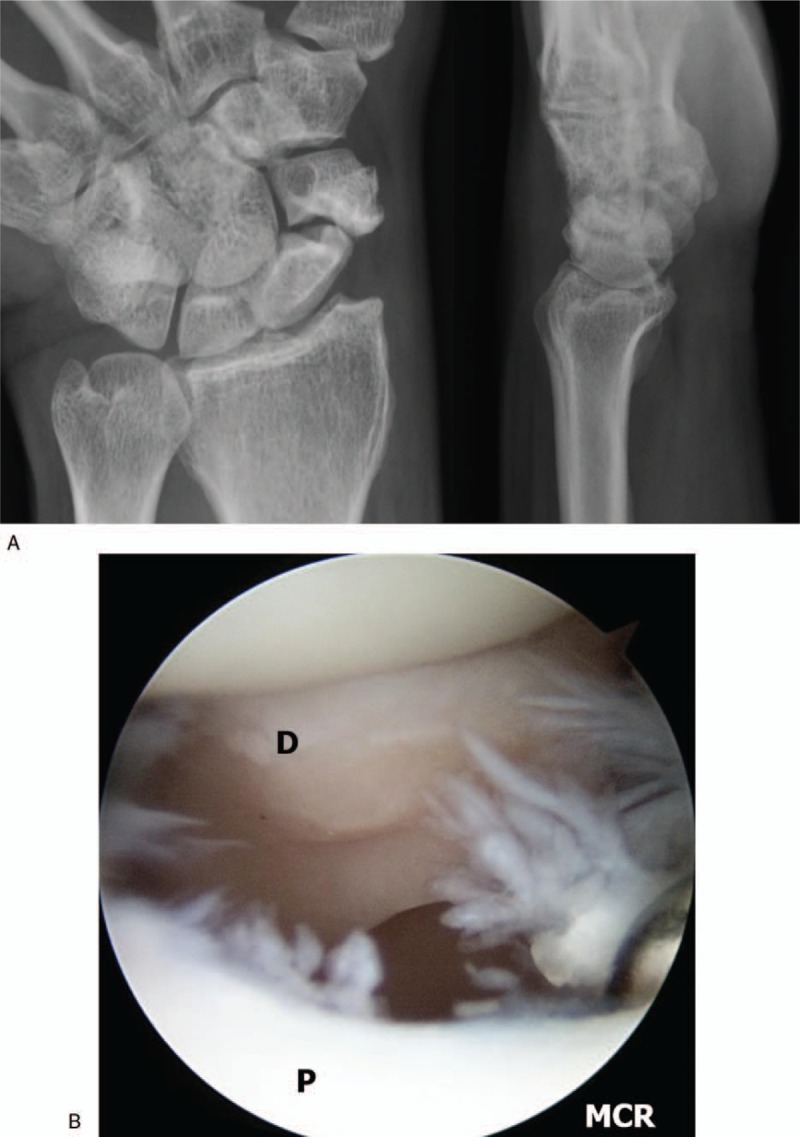
A 48-year-old male patient with nonunion of the left scaphoid fracture. Preoperative left wrist plain posteroanterior (PA) with ulnar deviation and lateral. (A) View showing nonunion at the waist of the scaphoid. (B) Same patient's left wrist, mid-carpal arthroscopy image of scaphoid nonunion site shows large gap and sclerotic margin of distal fragment. P = proximal fragment, PA = posteroanterior, D = distal fragment.

**Figure 4:**
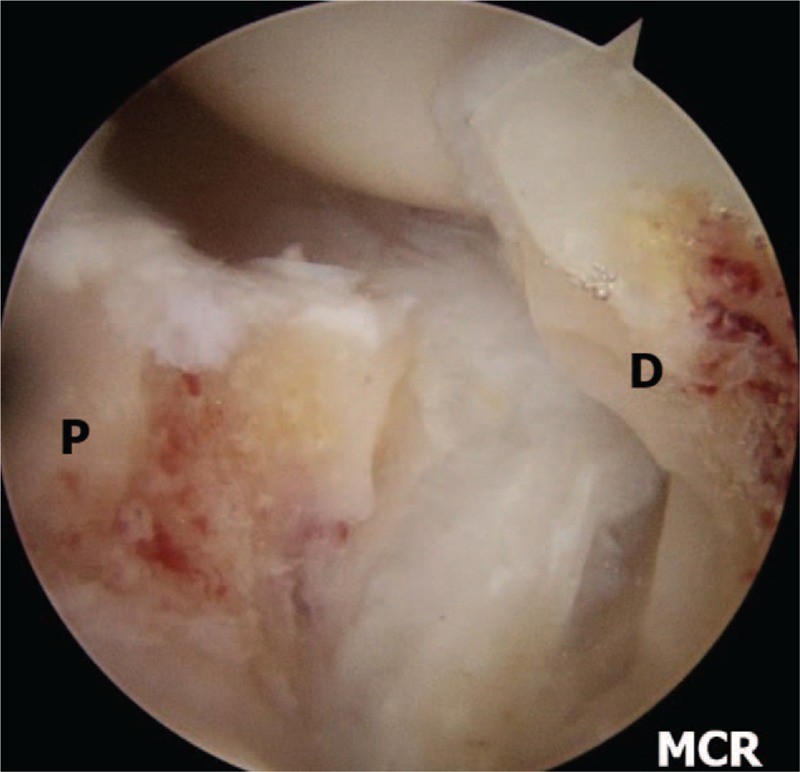
Left wrist, midcarpal arthroscopy image of nonunion site after debridement. Showing punctuate bleeding from proximal and distal fragments. P =  proximal fragment, D =  distal fragment.

**Figure 5:**
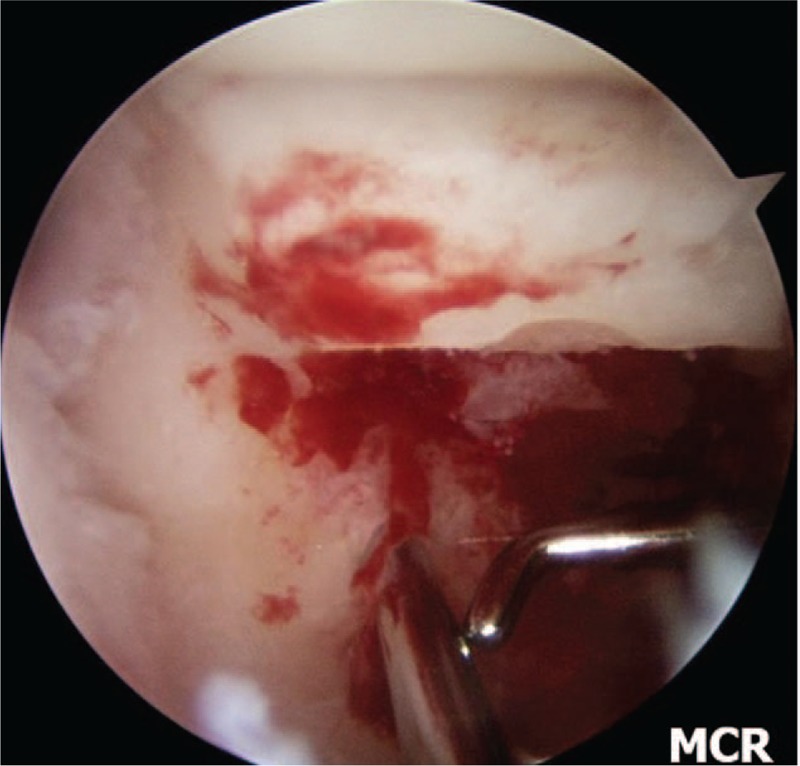
Arthroscopic view of provisional K-wire fixation.

We perform inspection on the radio-carpal joint at first. During arthroscopy, particular attention is paid to observe the status of the interosseous ligaments, articular cartilage, the presence of synovitis. We then transfer the arthroscope to the mid-carpal joint and examine the status of the articular cartilage and nonunion site (Fig. 3A and B). Both ends of the nonunion site are debrided and burred by switching the burr and shaver to the MCR and accessory portals until healthy looking cancellous bone with punctate bleeding can be seen (Fig. 4). We diagnosed the cases as AVN when no punctate bleeding was observed in the proximal fragment. After preparing the bone graft, we reduce the scaphoid under the C-arm image intensifier and a 1.2 mm K-wire is inserted percutaneously from the tubercle of the scaphoid to the proximal pole for provisional scaphoid fixation. In the presence of a dorsal intercalated segmental instability (DISI) deformity and extended lunate, we first flex the wrist to realign the extended lunate with the radius for deformity correction. The RL joint is then transfixed with a percutaneous 1.2 mm K-wire inserted from the dorsal distal radius. We then percutaneously fix the scaphoid with a 1.2 mm K-wire. We confirm the position of the K-wire under arthroscopic view before proceeding to the bone graft (Fig. 5). Cancellous bone graft is harvested from the iliac crest instead of distal radius using an open approach through a small incision. We want to get the volume of the harvested bone graft that has to be at least 3 to 5 times that of the defect because the graft needs to be tightly compressed into the defect to increase the strength of the graft. The bone graft is then cut into small chips using scissors.

**Figure 6:**
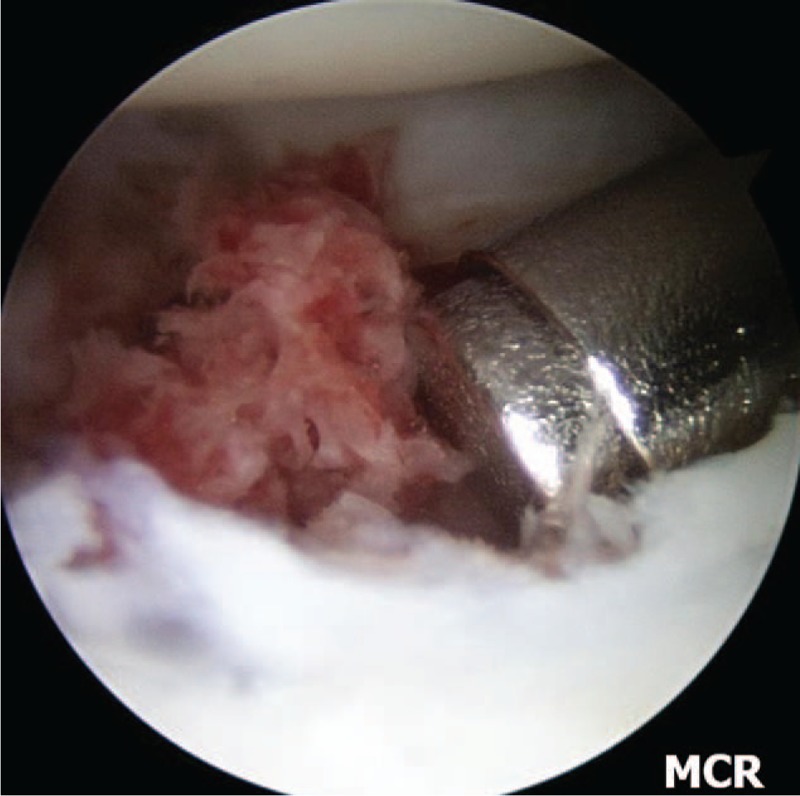
Same patient's left wrist, midcarpal arhroscopy images of percutaneous autogenous iliac cancellous bone grafting at the nonunion site using cannula and trocar.

**Figure 7:**
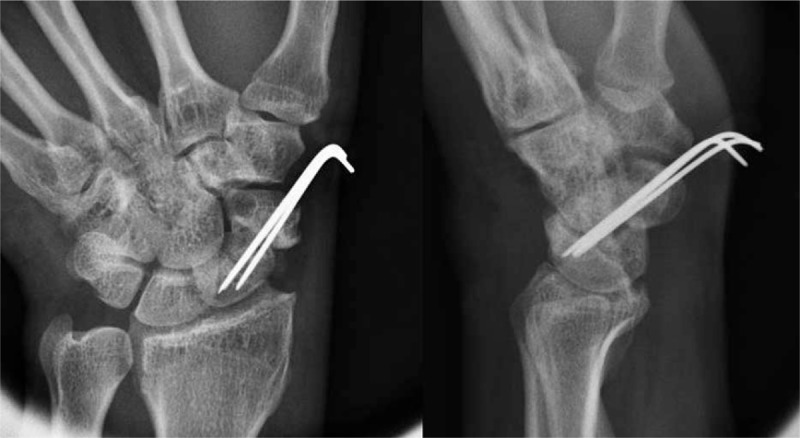
Immediate postoperative plain left wrist PA with ulnar deviation and lateral radiographs show internal fixation with K-wires and grafted bone at the nonunion site. PA = posteroanterior.

For bone grafting, an arthroscope is introduced in the MCU portal to continuously show the nonunion site, a custom-made 3.8 mm cannula is introduced to the nonunion site through the MCR portal, and cancellous chip bone is delivered to the entrance of the cannula. The bone graft is impacted with 3.2 mm trocar until a satisfactory volume of graft is achieved (Fig. 6). After completely filling the defect, routine surveillance of the joint is carried out to detect and remove any spilled bone graft material. We then routinely inject 1cc of fibrin glue (Greenplast Kit, Green Cross, Yongin Korea) into the surface of the graft substance. After arthroscopy, the wrist is taken out of traction. Definitive fixation with two 1.2 mm K-wires is performed under the C-arm image intensifier (Fig. 7). Additional SL K-wires fixation is performed to fix the unstable nonunion and is kept in place for 8 weeks. K-wires are then placed outside the skin.

**Figure 8:**
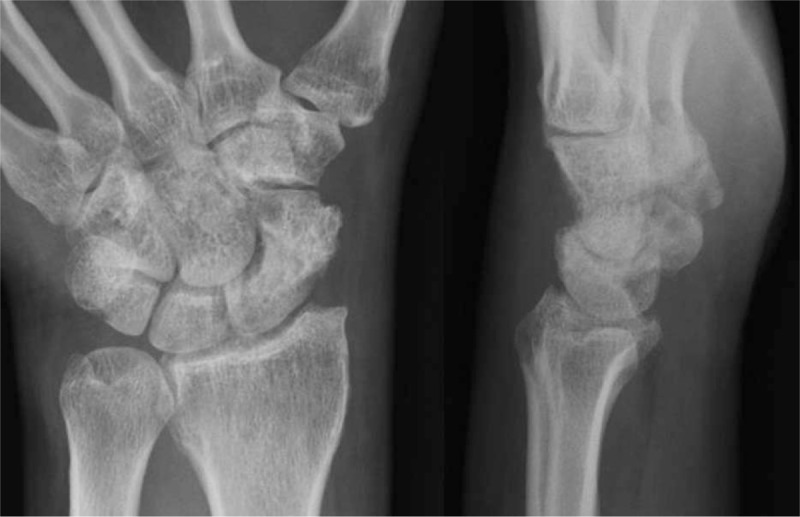
Postoperative 11 weeks K-wires removal plain left wrist plain PA with ulnar deviation and lateral radiographs shows complete bony union. PA = posteroanterior.

Postoperatively, in the case of stable nonunion without SL instability, the wrist is immobilized with a below elbow thumb spica splint. In the case of unstable nonunion with SL instability, the wrist is immobilized with an above elbow thumb spica splint for 2 weeks due to the protection of RL pinning which was removed at 2 weeks, after which we apply the below elbow thumb spica cast for 8 weeks. Plain radiographs are taken every week until bone union is achieved. When radiological union is confirmed, the K-wires are removed (Fig. 8).

## 3 Results

Union was achieved in 26 of 27 nonunions (96%). The average radiologic union time was 10 weeks (range, 7–14 weeks). Three patients with type 1-A tear of the TFCC were treated with debridement. The mean ROM of the wrist was improved to 178° (90.80% of that of the normal side), compared to a preoperative range of motion of 167° (83.38% of that of the normal side) (*P* < .05). The mean grip strength showed improvement from an average of 32.75 kg (81.84% of that of the normal side) preoperatively to 37.11 kg (89.65% of that of the normal side) at the last follow-up (*P* = .01). The average VAS score decreased from 6.38 (range, 3–10) preoperatively to 1.59 (range, 0–3) at the final follow-up (*P* < .05). The average modified Mayo wrist score improved from 60.19 preoperatively to 83.46 at the final follow-up (*P* < .05). When compared the 9 patients with SNAC and without SNAC, lower results were seen in patient with SNAC, and there were only statistically significant differences in ROM (*P* = .02). Given the average operation time of 212.5 minutes for the initial 10 cases and 152.5 minutes for the recent 10 cases, a learning curve is a necessary technique considering there was a significant statistical difference between the 2 (*P* < .05). There was one failure in our series. The patient required revision with an open corticocancellous bone graft. Union was obtained at 14 weeks of postoperation, and the patient was pain free and returned to daily life (Table 4).

**Table 4:**
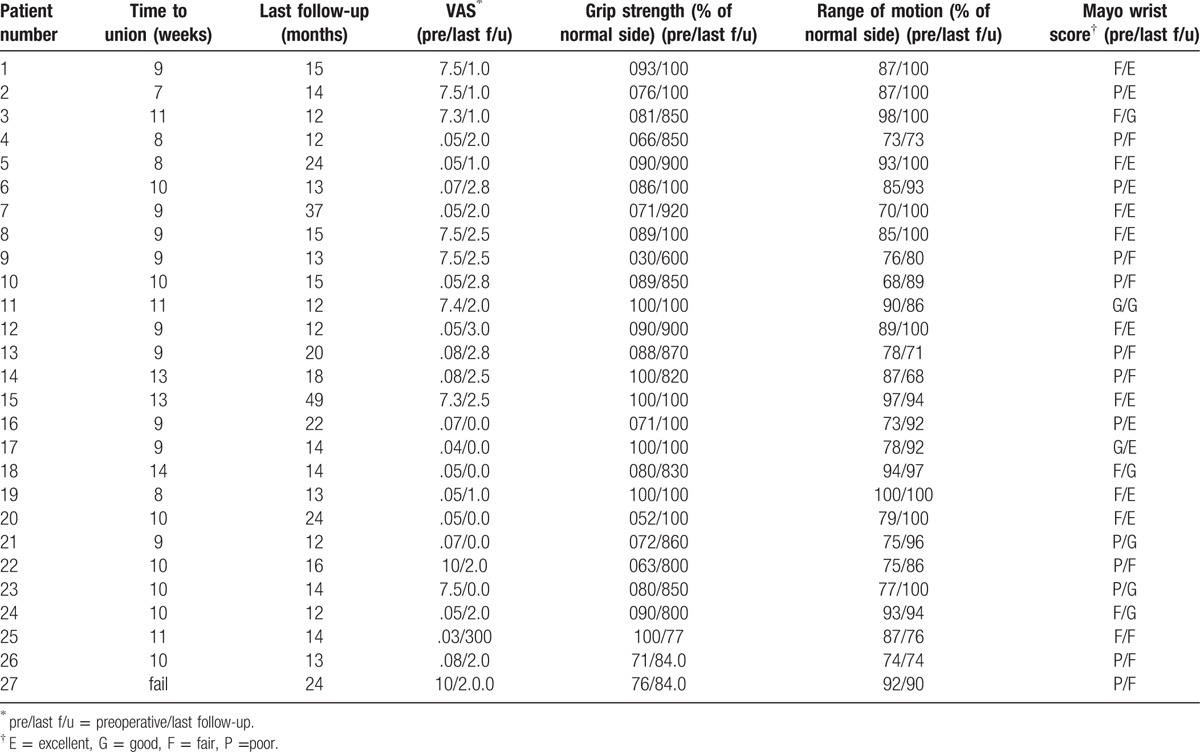
Demography of the patients.

## 4 Discussion

Scaphoid nonunions have reported treatment failure ranges from 25% to 45% and still it gives a difficult challenge for even the most experienced wrist surgeons.^[4–6,22]^ The treatment for scaphoid nonunion varies, but maintaining blood supply, debridement of necrotic bone and scar tissue, exposure of healthy well-vascularized cancellous scaphoid bone, fracture reduction, bone grafting, and rigid internal stabilization are critical requirement.^[7,23]^ The advantage of open bone grafting for scaphoid nonunion is that it offers direct visualization of the nonunion site for the freshening, correction of accompanied deformities, and reduction. However, open method occasionally results in devitalization of the nonunion site due to unnecessary stripping making further complications.^[7,8,14]^ For these reasons, the application of arthroscopic-assisted percutaneous and minimally invasive procedures for scaphoid nonunion has recently been attempted.^[9,13–16]^ And several authors reported 96 to 100% of bone union rate.^[13,24–26]^

The advantages of arthroscopic-assisted percutaneous and minimally invasive procedures can avoid carpal ligament injury because they do not require an open arthrotomy and can preserve as much of the tenuous blood supply of scaphoid as possible.^[9–16]^ The minimal disturbance of the vascularity of the scaphoid may contribute to the high union rate and a relatively short period for bone union compared to open treatment. We achieved bone union at an average of 10 weeks in 26 cases. Slade et al^[24]^ reported union at an average of fourteen weeks in all 15 patients with grade 2 to 3 nonunion using a dorsal approach with arthroscopy and without bone grafting. Even though our 13 cases are more than grade 4 according to the Slade and Geissler's scaphoid classification, we believe that our average bone union time is much shorter because percutaneously iliac cancellous bone grafting was performed in healthy cancellous scaphoid bone made by debridement. Although carpal ligament injury was present in 17 cases and SNAC in 9 cases among the patients who had get the bone union, the range of motion (ROM) of patients at the final follow-up was improved in 14 cases. We believe this is because we avoided additional injury of the joint capsule and carpal ligament by using a minimally invasive method. Slade et al^[9,22]^ described that early degenerative changes to the carpus permit the repair of the scaphoid nonunion with open debridement, interpositional corticocancellous bone graft or vascularized bone graft, and rigid fixation and treatment of the local arthritis with radial styloidectomy. Stage I SNAC is defined as an isolated distal styloid arthritis due to a styloid-scaphoid impingement. In cases with early SNAC changes, fixation of the nonunion is not contraindicated. We hypothesized that if a fracture is well reduced, a styloid-scaphoid impingement will not occur, arthritis will not progress, and pain will not be caused. Radial styloidectomy was not performed on patients with degenerative arthritis in the initial surgery because it can be conducted if the pain occurs due to styloid-scaphoid impingement during a follow-up after bone union is initially achieved. However, a sufficient arthroscopic debridement was performed including synovectomy. We think it was because an additional styloid-scaphoid impingement did not occur as nonunion recovered well, and an additional damage on joint capsule or ligament was avoided by using a minimally invasive method. Since it is still controversial, further studies on more cases and longer follow-up are deemed necessary. No punctate bleeding was observed in the proximal fragment in 3 cases, and we diagnosed these cases as AVN. However, we could achieve bone union in all cases. We believe this is also due to the minimal disturbance of the scaphoid blood supply. Accurate diagnosis and treatment for intra-articular pathology and cartilage are possible at the same time. We identified SL ligament injuries in 17 (63%) patients, LT ligament injuries in 3 patients and 3 type I-A injuries of TFCC.

We used K-wires for fixation of scaphoid. Chen et al^[27]^ reported a 100% union rate in 39 patients treated for scaphoid nonunion with corticocancellous bone graft and multiple, divergent K-wire fixation. Takami et al^[28]^ reported a 98% union rate in 43 patients using corticocancellous bone graft combined with K-wire fixation. We achieved the bone union with 1.2 mm K-wires in 26 patients. In addition, if we cannot achieve bone union, the K-wire is a more useful technique in terms of preparing for the secondary operation.

Serial plain radiographs have typically been used to demonstrate the union of scaphoid fracture by identification of the trabeculae crossing the fracture line or sclerosis at the fracture line. However, the general radiographic definition of union is debatable and the interobserver agreement for the radiographic diagnosis of union of scaphoid fracture was poor. Second it is difficult to obtain good quality radiographs in the same plane as the fracture line. Third various patterns of partial union obscure interpretation. Therefore, the evaluation of radiographs for scaphoid union is less accurate than computerized tomography (CT) scan.^[29–32]^ Even when using CT scan interpretation of the union can be a challenge. But CT is a much more reliable tool than plain radiographs in the evaluation of scaphoid union and deformity.^[33–35]^

Several circumstances precluded the use of arthroscopic bone grafting for scaphoid nonunion. First, if a surgeon lacks experiences in wrist arthroscopy and arthroscopic anatomy, he should not attempt this technique. Secondly, a severely destroyed wrist combined with scaphoid AVN precludes arthroscopic bone grafting. Finally, significant arthrofibrosis of the wrist can cause difficulty when performing arthroscopic bone grafting. We will perform arthroscopic bone grafting for scaphoid nonunion and delayed union, with the exception of the above mentioned limitations.

Our study has a number of limitations. First, a small number of patients were included. Second, a direct comparison of our result is difficult because the results of the operation method used had not been previously described. Third, the observers were not independent from the clinical and radiographic outcomes. Fourth, the authors used plain radiographs to assess the scaphoid union. However, CT scans are far more accurate in assessing the scaphoid union, and it is a significant limitation of this study.

## 5 Conclusion

Arthroscopic bone grafting and percutaneous K-wires fixation is an effective treatment method for a scaphoid nonunion and has advantages such as allowing through assessment and a comprehensive management approach for scaphoid nonunion in a minimally invasive manner and this method can also be used for the scaphoid nonunion with SNAC stage I.

## Author contributions

**Data curation:** S.-H. Woo.

**Funding acquisition:** M. Lee.

**Investigation:** M. Lee, K.-W. Choi, P.C. Ho.

**Methodology:** P.C. Ho.

**Resources:** P.C. Ho.

**Supervision:** Y.-K. Lee.

**Validation:** Y.-K. Lee.

**Visualization:** K.-W. Choi.

**Writing – original draft:** Y.-K. Lee, M. Lee.

**Writing – review & editing:** Y.-K. Lee, M. Lee.
